# Overexpression of a MADS-Box Gene from Birch (*Betula platyphylla*) Promotes Flowering and Enhances Chloroplast Development in Transgenic Tobacco

**DOI:** 10.1371/journal.pone.0063398

**Published:** 2013-05-17

**Authors:** Guan-Zheng Qu, Tangchun Zheng, Guifeng Liu, Wenjie Wang, Lina Zang, Huanzhen Liu, Chuanping Yang

**Affiliations:** 1 State Key Laboratory of Forest Tree Genetics and Breeding, Northeast Forestry University, Harbin, China; 2 Key Laboratory of Forest Plant Ecology, Northeast Forestry University, Harbin, China; Ghent University, Belgium

## Abstract

In this study, a MADS-box gene (*BpMADS*), which is an ortholog of *AP1* from *Arabidopsis*, was isolated from birch (*Betula platyphylla*). Transgenic *Arabidopsis* containing a *BpMADS* promoter::GUS construct was produced, which exhibited strong GUS staining in sepal tissues. Ectopic expression of *BpMADS* significantly enhanced the flowering of tobacco (35S::BpMADS). In addition, the chloroplasts of transgenic tobacco exhibited much higher growth and division rates, as well rates of photosynthesis, than wild-type. A grafting experiment demonstrated that the flowering time of the scion was not affected by stock that overexpressed *BpMADS*. In addition, the overexpression of *BpMADS* resulted in the upregulation of some flowering-related genes in tobacco.

## Introduction

In plants, vegetative and reproductive development occur postembryonically in the meristem, a small group of undifferentiated cells. Shoot apical meristems (SAMs) can develop into either shoots or flowers. The transition from the vegetative phase to the reproductive phase is controlled by complex genetic networks. Five genetically defined pathways, including the vernalization, photoperiod, gibberellin, autonomous, and endogenous pathways, control flowering [1]. *Arabidopsis* plants with mutations in three genes, including *CO*, *FCA*, and *GA1*, which function in the long-day, autonomous, and gibberellin pathways, respectively, never flower under long- or short-day conditions. This indicates that the three pathways impaired by these mutations are absolutely required for flowering under long- or short-day conditions [2]. Signals from different pathways interact to regulate floral development through the action of flowering pathway integrators, such as FT and SOC1. Integrators act upstream of floral meristem identity genes, such as *AP1* and *LFY*, to determine the fate of the SAM [3], [4]. In *Arabidopsis*, AP1 converts inflorescence shoot meristems into floral meristems, and ectopic *AP1* expression can dramatically reduce the time to flowering [5]. In addition, *LFY* expression in *Arabidopsis* is sufficient to determine floral fate in lateral shoot meristems of *Arabidopsis* [6]. In strong *ap1* mutants of *Arabidopsis*, sepals and petals fail to develop, and secondary flowers develop in the axils of sepals that have been converted into bracts [7], [8].

In this study, we cloned a MADS-box gene from *Betula platyphylla* Suk (*BpMADS*), which is an ortholog of *AP1* in *Arabidopsis*. The ectopic expression of *BpMADS* in tobacco (*Nicotiana tabacum*) not only promoted the transition to flowering, but it also enhanced the division and growth of the chloroplast.

## Materials and Methods

### Isolation of *BpMADS*

We previously constructed a SSH cDNA library from the female inflorescence of *Betula platyphylla* Suk (unpublished data). The partial sequence of *BpMADS* (GenBank No. EE284583) showed high levels of homology (73%) with AP1 in Arabidopsis according to the Blast analysis in GenBank. The full-length cDNA for *BpMADS* was cloned from total RNA of birch inflorescences using the SMART™ RACE cDNA Amplification Kit (Clontech, Palo Alto, CA, USA), according to the manufacturer’s instructions. The primers used to amplify the open reading frame (ORF) of *BpMADS* were as follows: MADS-Forward: (ATGGGGAGGGGTAGGGTTCAGCT) and MADS-Reverse: (TCACGTGGCAAAGCATCCAAGGT). The deduced BpMADS cDNA protein was identified using Expasy tools (http://www.expasy.org/tools).

### Quantitative Real-time PCR Analysis

To detect the expression of *BpMADS*, total RNA was isolated from male inflorescences, leaf buds, and young leaves of birch tree (*Betula platyphylla* Suk) that were exposed to 6 h of light or 6 h of dark in a greenhouse, respectively. For quantitative real-time PCR analysis (Q-PCR), a PrimeScript RT Reagent Kit (TaKaRa, Dalian, China), SYBR Premix EX Taq II (TaKaRa), and an MJ Opticon 2 System (Bio-Rad, Hercules, CA, USA) were employed following the manufacturer’s instructions. Gene-specific primers were used to quantify the *BpMADS* transcripts, and 18 s ribosomal RNA was used as an internal reference. To estimate the transcript levels of flowering-related genes in transgenic and wild-type tobacco with Q-PCR, aerial parts of 20-day-old transgenic and wild-type tobacco plants were harvested before flower buds were visible. Specific primers for flowering-related genes were used for Q-PCR, and the actin gene (GenBank No. JQ435884) was used as an internal reference. All primers employed for Q-PCR are shown in Table 1. Each reaction was conducted in triplicate to ensure the reproducibility of results. Expression levels were calculated from the cycle threshold using the delta-delta CT method [9].

**Table 1:** Primers employed in quantitative real-time PCR.

Plant species	Gene	Sequence
Birch	18S-F	ATCTTGGGTTGGGCAGATCG
	18S-R	CATTACTCCGATCCCGAAGG
	BpMADS-F	CTAGTTGCAGCTGATTCTGAAGG
	BpMADS-R	CCTTTCTCTGAAGCTGCGAG
Tobacco	NtActin-F	TGTGTTGGACTCTGGTGATG
	NtActin-R	CGCTCGGTAAGGATCTTCATC
	NsMADS3-F	ACAACTCAAACTCAGGGCG
	NsMADS3-R	TGTAGTTATTCACACTGGGCC
	NtMADS4-F	GGAAGACTCAAAACATGCTGG
	NtMADS4-R	TGACAATCGAAGTGGAACTCG
	NtMADS5-F	GCATGGGTACAAGGAATCAAG
	NtMADS5-R	TCCTACAACCACATCCTTGC
	NtMADS11-F	AAGAAAGGGAGAAAGAGCTGG
	NtMADS11-R	GGGCTGTGACAAAACGAATG
	NtFUL-F	GCTCTTAAACACATTCGCTCAAG
	NtFUL-R	CAACTCTTTCTCCCTCTCCTTC
	NtSOC1-F	TGGCCTTCAACAGAGACAAG
	NtSOC1-R	ACGCCTTATTCTGCACTCG
	NFL2-F	AATCAGGTGTTCAGGTACGC
	NFL2-R	TCGCTCCTTGAAAGCTCTTC

### Generation of Transgenic *Arabidopsis*

A 1.8-Kb fragment upstream of the transcription start site of *BpMADS* was amplified by PCR using genomic DNA from birch with a Genome Walking Kit (Takara, Dalian, China), according to the manufacturer’s instructions. The DNA fragment was inserted into the vector pCAMBIA-1301 to obtain a GUS fusion vector, in which the 35S promoter upstream of GUS was deleted and replaced with the *BpMADS* promoter. The resulting construct was introduced into *Agrobacterium tumefaciens* strain EHA105 and transformed into *Arabidopsis thaliana* (ecotype Col-0) plants using the floral dip method [10].

### Northern Blot Analyses

To detect the expression of exogenous *BpMADS*, 10 µg of total RNA from young leaves of 20-day-old tobacco plant was separated on 1% agarose denaturing formaldehyde gel, transferred to a Hybond-N^+^nylon membrane and fixed by UV cross-linking (254 nm, 8 min). Hybridization and detection were conducted following the manufacturer’s instructions (DIG Northern Starter Kit, Roche, Mannheim, Germany).

### Subcellular Localization Analysis

The cDNA encoding the ORF of *BpMADS* was cloned into the vector pTH2 to generate the BpMADS-GFP gene fusion driven by the CaMV 35S promoter as described by Niwa [11]. The BpMADS-GFP construct was transformed into onion epidermal cells by particle bombardment (Bio-Rad PDS-1000/He System, USA). The transient expression of the BpMADS-GFP fusion protein was observed through a Zeiss confocal microscope.

### Generation of Transgenic Tobacco Plants Overexpressing *BpMADS*

To constitutively express in *BpMADS* in tobacco, *BpMADS* was cloned into the *Xba*I and *Sac*I sites of the pROKII vector under the control of the CaMV 35S promoter and the NOS terminator. The resulting vector, pROKII-BpMADS, was transferred into *Agrobacterium tumefaciens* strain EHA105 using the freeze–thaw transformation method [12]. Transgenic tobacco plants (*Nicotiana tabacum* cv. ‘Havana SRI’) were obtained by *Agrobacterium*-mediated transformation as described by Horsch et al. [13]. Tobacco transformants were selected on differentiation medium (MS medium supplemented with 20 g l^−1^ sucrose, 0.1 mg l^−1^ 1-Naphthylacetic acid [NAA], 0.5 mg l^−1^ N6-Benzyladenine [6-BA], and 8 g l^−1^ agar) containing 60 mg l^−1^ kanamycin (Km). Tobacco seedlings were germinated and grown in pots containing soil in a growth room at 22±2°C under lights with a photon flux density of 200 µmol m^−2^ s^−1^, with a 16 h light/8 h dark cycle.

### Histological Microscope Observations

To observe the cell morphology of tobacco leaves, plant tissue samples mounted on scanning electron microscope (SEM) stubs were frozen in liquid nitrogen for several minutes and quickly observed under a scanning electron microscope (FEI Quanta 200).

To observe chloroplasts, leaf samples were fixed in 5% glutaraldehyde solution with 0.05 M phosphate at 4°C and post fixed in 2% OsO_4_ for 2 hours. The samples were dehydrated in ethyl alcohol and embedded in epoxy resin. For transmission electron microscopy (TEM) observation, pale golden sections of fixed samples were loaded onto 100-mesh copper grids coated with Formvar (1% in ethylene dichloride), stained with uranyl acetate and lead citrate, and viewed with TEM.

For histological observation of vegetative apices of tobacco, 15-day-old seedlings were collected and fixed by immersion in a formalin:acetic acid:ethanol:water solution (FAA, 1∶1:9∶9 v:v) at room temperature for 48 h and stored in 70% ethanol at 4°C. The fixed material was then stained with hematoxylin solution (Ehrlich) for 2 days. After dehydration through an ethanol series (30, 50, 70, 85, 95, and 100% at 2 h for each concentration), the samples were embedded in paraffin wax (58–60°C). Longitudinal semi-sections (8 µm) were produced from fixed tobacco apices using a microtome (HM 340 E; Microm International GmbH, Walldorf, Germany) and fixed onto glass slides. The sections were dewaxed in xylene for 5–10 min and observed under a light microscope (Docuval, Carl Zeiss, Germany).

### Histochemical Staining for Lignin

For phloroglucinol-HCl staining, hand-cut sections of 4-week-old tobacco stems were collected and fixed by immersion in FAA fixation liquid at room temperature for 48 h and stained with 1% phloroglucinol in 95% ethanol and concentrated HCl (2∶1, v:v) for 5 min, followed by observation under a light microscope.

### Histochemical Staining for Starch

For starch-iodine staining, young leaves of 3-week-old tobacco plants subjected to 3 h of light treatment were harvested and fixed in FAA fixation liquid for 24 h. After chlorophyll extraction, the leaves were incubated in iodine solution for 20 min and washed with deionized water. The leaves, which contained iodine-stained starch, were photographed with a digital camera.

### Histochemical Analysis for GUS Activity

Histochemical analysis of GUS activity was performed as described by Jefferson et al. [14]. The plant material was collected and immediately incubated in GUS staining buffer (100 mM sodium phosphate pH 7.0, 1 mM 5-bromo-4-chloro-3-indolyl-β-D-glucuronide, 0.5 mM potassium ferrocyanide, 0.5 mM potassium ferricyanide, 10 mM Na_2_EDTA, and 0.1% Triton X-100) for 16 h at 37°C. The stained tissues were then rinsed with a graded ethanol series until pigments such as chlorophyll were completely cleared. The samples were then photographed with a camera mounted on a Carl Zeiss laser scanning microscope.

### Chlorophyll Content Analysis

The chlorophyll content of leaves was measured using the DMSO method as described by Barnes et al. [15] and Shinano et al. [16].

### Measurement of Photosynthetic Rates

Leaf photosynthesis was measured with a LI-6400 portable photosynthesis system (LiCor, Nebraska, USA). After stabilization in a greenhouse for 1 hour, the leaf photosynthetic rate under saturated light was measured, and photosynthetic active radiation (PAR) was maintained at 1,500 µmol m^−2^ s^−1^ using an LED red-blue light source.

### Grafting Experiment

In this experiment, 25-day-old tobacco seedlings, which contained three leaves, were used as stock, and 14-day-old seedlings were used as scion. A classic wedge-shaped/slit grafting technique was employed, and the site of the union was wrapped with Parafilm. The plants were covered with plastic bags to maintain a relative humidity level of 80%. The plants were grown for a total of 10 days in 80% humidity.

## Results

### Isolation of *BpMADS*

The cDNA for the *BpMADS* ORF is 732 bp in length from the ATG start codon to the TGA stop codon. This cDNA encodes a predicted polypeptide of 243 amino acids with a molecular weight of 28.01 kDa and a pI of 8.99. The sequence was deposited in GenBank under accession number JX565468. The predicted BpMADS polypeptide exhibits high identity at the amino acid level (74%) with AP1 (AT1G69120) in *Arabidopsis* and extremely high identity (97%) with BpMADS3 (GenBank No. X99653) from *Betula pendula* [17].

### Subcellular Localization Analysis of *BpMADS*

**Figure 1:**
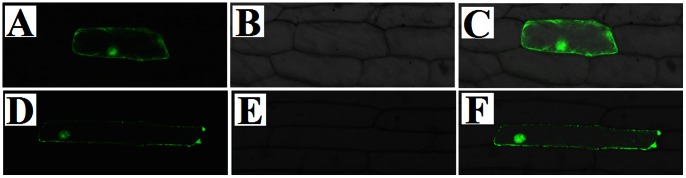
Subcellular localization analysis of *BpMADS*. The *BpMADS-GFP* fusion (and *GFP* alone) were transiently expressed under the control of the CaMV 35S promoter in onion epidermal cells and observed under a confocal microscope. The photographs were taken in dark field for green fluorescence localization (A, D), in bright light to examine cellular morphology (B, E), and in combination (C, F). (A, B, C) Transient expression of GFP in the control (D, E, F) Expression of the BpMADS-GFP fusion protein.

The subcellular localization of the BpMADS protein was examined by introducing the BpMADS-GFP fusion protein into onion epidermal cells by particle bombardment. GFP from the control plasmid (lacking *BpMADS*) localized to the cytoplasm and the nucleus (Figure 1A–1C). BpMADS-GFP fluorescence in transformed onion cells was observed in the cytoplasm and the nucleus (Figure 1D–1F). In a previous study examining the expression of the *AP1* ortholog of *Arabidopsis*, expression of the C-terminal AP1-GFP appeared to occurred exclusively in the nucleus, while the N-terminal GFP:AP1 fusion produced a mainly cytoplasmic signal [18]. In present study, transformed onion epidermal cells expressing C-terminal BpMADS:GFP produced both cytoplasmic and nuclear signals.

### Expression Profiles of *BpMADS*

**Figure 2:**
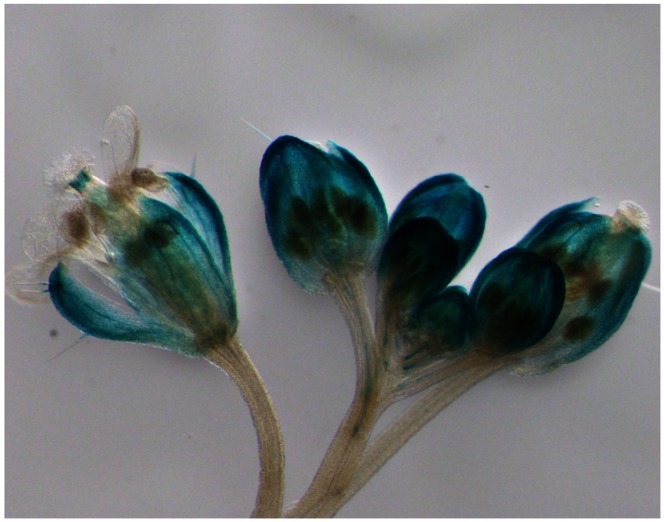
GUS staining of transgenic *Arabidopsis* inflorescence. The inflorescence was sampled from a 30-day-old seedling of an *Arabidopsis* transformant (containing the *BpMADS* promoter driving the GUS reporter gene).

Transgenic *Arabidopsis* plants containing the *BpMADS* promoter fused to *GUS* (BpMADS::GUS) were examined by histochemical analysis. Strong GUS activity was detected in young sepals of the transformants (Figure 2). In *Arabidopsis*, AP1 RNA is uniformly expressed in young flower primordia, and later becomes localized to sepals and petals, which suggests that AP1 acts locally to specify the identity of the floral meristem and to determine sepal and petal development [19]. No GUS activity was detected in the petals of BpMADS::GUS *Arabidopsis* plants, suggesting that there are differences in the expression profiles of *AP1* in *Arabidopsis* and its ortholog in birch.

The male inflorescences of birch are visible in Northeast China in July. We therefore examined the expression levels of *BpMADS* in male inflorescence buds and leaves in July in plants exposed to light or dark conditions using Q-PCR (Table 2). *BpMADS* exhibited a high level of expression in male inflorescences and extremely low levels of expression in leaves, which is consistent with the results of Elo et al. [17]. In addition, our experiment demonstrated that light treatment increases the expression of *BpMADS* in birch.

**Table 2:** Relative expression of *BpMADS* in birch.

	Inflorescence	Leaves
Dark	10089.57±1283.35	0.63±0.13
Light	4623.23±200.57	0.13±0.02

Young leaves and inflorescences of birch treated with 6 h of light or 6 h of dark. Values are expressed as mean ± S.D. (n = 3 samples for each test).

### Modulation of Flowering Time by Constitutive Expression of *BpMADS*

**Figure 3:**
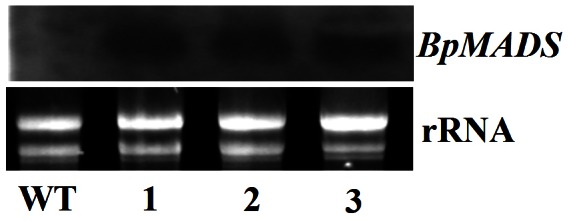
Northern blot analysis of *BpMADS* expression in transgenic tobacco. Leaves were sampled from wild-type and transgenic plants. WT, wild-type; 1–3, three 35S::BpMADS lines (MADS-1, MADS-2, and MADS-3).

**Figure 4:**
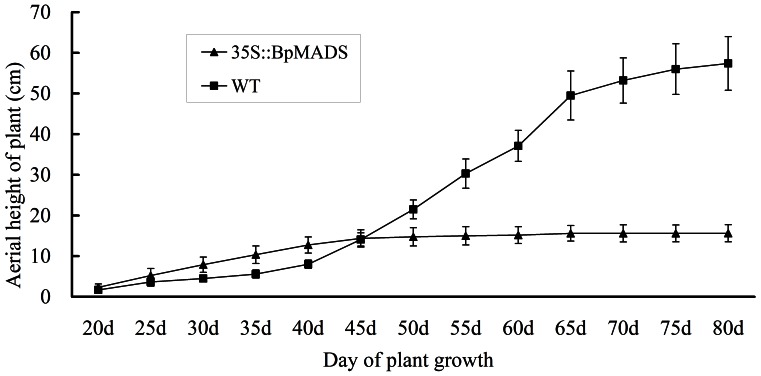
Plant height of transgenic and wild-type tobacco at different time points. The tobacco seedlings were grown in a greenhouse. Each test was repeated with nine seedlings (n = 9). Nine seedlings from homozygous 35S::BpMADS lines (MADS-1, MADS-2 and MADS-3) were selected at random. Error bars indicate SD.

**Figure 5:**
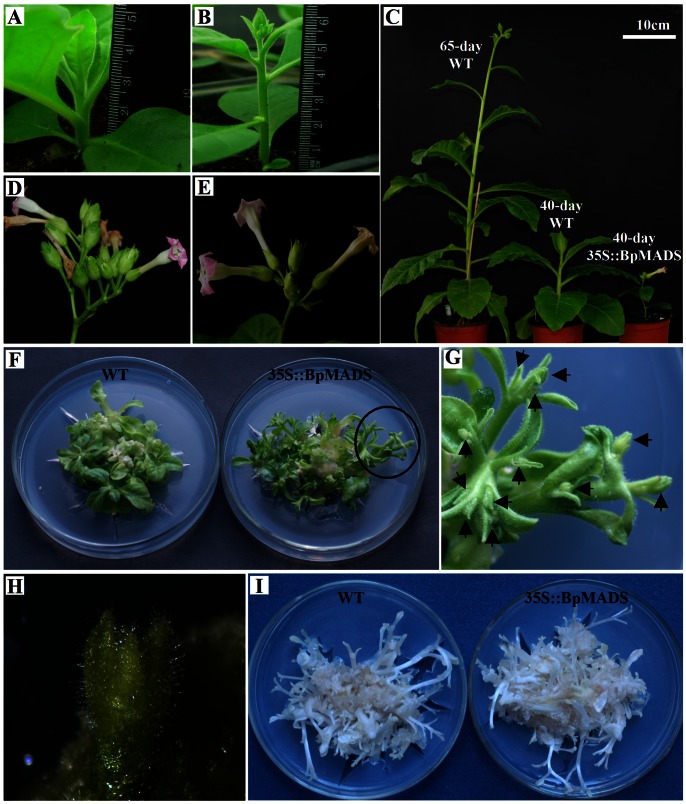
Effects of *BpMADS* overexpression on flowering in transgenic tobacco. (A) 28-day-old wild-type and (B) 35S::BpMADS seedlings. (C) phenotype of wild-type and 35S::BpMADS seedlings at various time points; 65-day-old wild-type and 40-day-old 35S::BpMADS were in bloom. (D, E) Flower and fruit phenotype of wild-type (D) and 35S::BpMADS (E) plants. (F) Wild-type and 35S::BpMADS leaf explants were cultured on differentiation media (MS medium supplemented with 20 g/L sucrose, 0.1 mg/L NAA, 0.5 mg/L 6-BA, and 8 g/L agar) for 30 days under normal light conditions; no flower buds were found in the wild-type. (G) Close-up view of the circled area in (F); arrows indicate flower buds. (H) Rudimentary flower bud formed directly from a leaf disk on differentiation media. (I) Wild-type and 35S::BpMADS leaf explants were cultured on differentiation media for 30 days in the dark; no flower buds developed on wild-type or 35S::BpMADS plants.

Tobacco was transformed with *BpMADS* under the control of the constitutive CaMV 35S promoter. Three homozygous (T3 generation) 35S::BpMADS tobacco lines (MADS-1, MADS-2, and MADS-3) were selected for subsequent analysis. Northern blot analysis confirmed that these lines overexpressed exogenous *BpMADS*, while wild-type tobacco did not (Figure 3). The transgenic plants (35S::BpMADS) exhibited much earlier stem initiation than the wild-type. However, the transformants were dwarfed, with a final aerial height of less than 15 cm, while the final aerial height of wild-type plants was greater than 50 cm after more than 80 days of growth (Figure 4). In addition, the transgenic plants flowered considerably earlier than wild-type plants, indicating that the overexpression of *BpMADS* is sufficient to promote the transition from vegetative to reproductive development (Figure 5A–5C). Flower buds were first visible in the transformants only four weeks after sowing, when the aerial heights of the plants were under 4 cm. Under long-day conditions (with a 16 h light/8 h dark cycle), all transformants flowered less than 40 days after sowing, whereas the wild-type plants flowered 65 days after sowing (Figure 5C). The transformants were dwarfed and produced fewer than 10 leaves before flowering. By contrast, wild-type plants produced more than 15 leaves before flowering. However, there was no significant variation in either flower or fruit morphology, or fertility levels, between transgenic and WT plants, although the transgenic plants developed lower numbers of flower buds and fruits than the wild-type (Figure 5D and 5E).

When cultured on differentiation medium, transgenic leaf disks exhibited a strikingly different morphology from those of wild-type. All of the normal apical and lateral shoots developed into terminal rudimentary flowers, with petals, stamens, and some floral organs that were not well developed, while no flower buds were present on the wild-type shoots at the same time point (Figure 5F and 5G). Some flower buds formed directly on transgenic leaf disks, instead of forming from shoot meristems that were transformed into floral meristems (Figure 5H). When leaf explants were cultured on differentiation medium in the dark, there was no significant difference in phenotype between transgenic and wild-type plants, with no flower buds generated on transgenic leaf disks. These results suggest that the accelerated conversion to flowering caused by the overexpression of *BpMADS* requires the presence of light (Figure 5I).

### Altered Morphology and Development of 35S::BpMADS Plants

**Figure 6:**
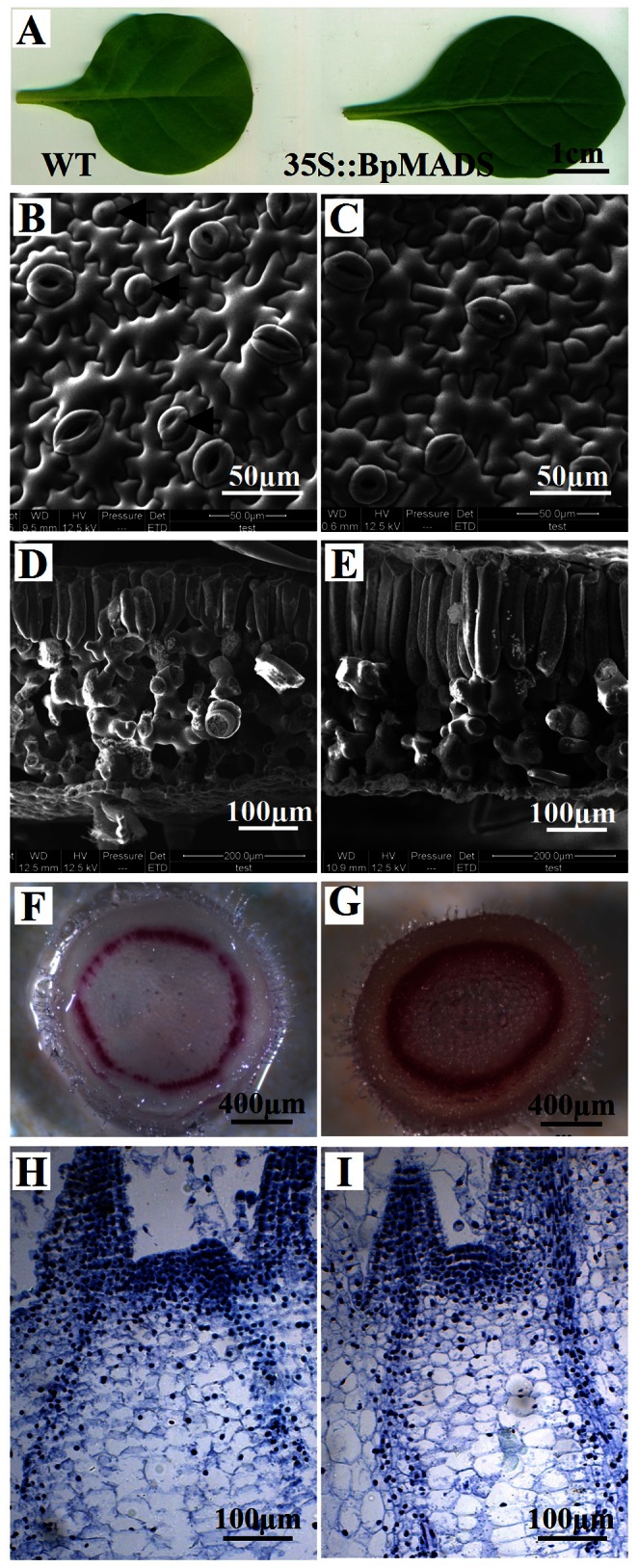
Microscopic observation of tobacco. (A) The fourth true leaves from 25-day-old wild-type and 35S::BpMADS seedlings. (B, C) SEM observation of upper-epidermal cells in the third true leaves of 20-day-old wild-type (B) and 35S::BpMADS (C) seedlings; arrows indicate immature guard cells. (D, E) SEM observation of cross sections of third true leaves from 20-day-old wild-type (D) and 35S::BpMADS (E) seedlings. (F, G) Hand-cut sections of 4-week-old wild-type (F) and 35S::BpMADS (G) tobacco stems treated with phloroglucinol-HCl for lignin staining. (H, I) Histological observation of apical shoots from 15-day-old wild-type (H) and 35S::BpMADS (I) tobacco seedlings.

In addition to affecting the induction of flowering, the overexpression of *BpMADS* had other affects on the phenotype of the transgenic lines. As shown in Figure 6A, the 35S::BpMADS plants exhibited profound changes in the shapes of the fourth and subsequent true leaves, with a higher ratio of length/width in the transgenic plants than in the wild-type. SEM results indicate that guard cells in young transgenic leaves developed earlier than those in wild-type plants (Figure 6B and 6C). In addition, the palisade cells of transgenic plants were large and well developed and were arranged more closely together than those of the wild-type (Figure 6D and 6E). Phloroglucinol reacts with coniferaldehyde groups in lignin, resulting in staining; the intensity of staining reflects the total lignin content [20]. The results of phloroglucinol staining indicate that the stem of transgenic tobacco contained much more lignin than wild-type, suggesting that the overexpression of *BpMADS* accelerates the formation of lignin (Figure 6F and 6G). Histological observation revealed that the indeterminate meristems of transgenic seedlings did not develop as floral meristems 15 days after sowing. However, wild-type plants had bigger meristems than transgenic plants (Figure 6H and 6I).

### Chlorophyll Content and Photosynthetic Analysis

**Figure 7:**
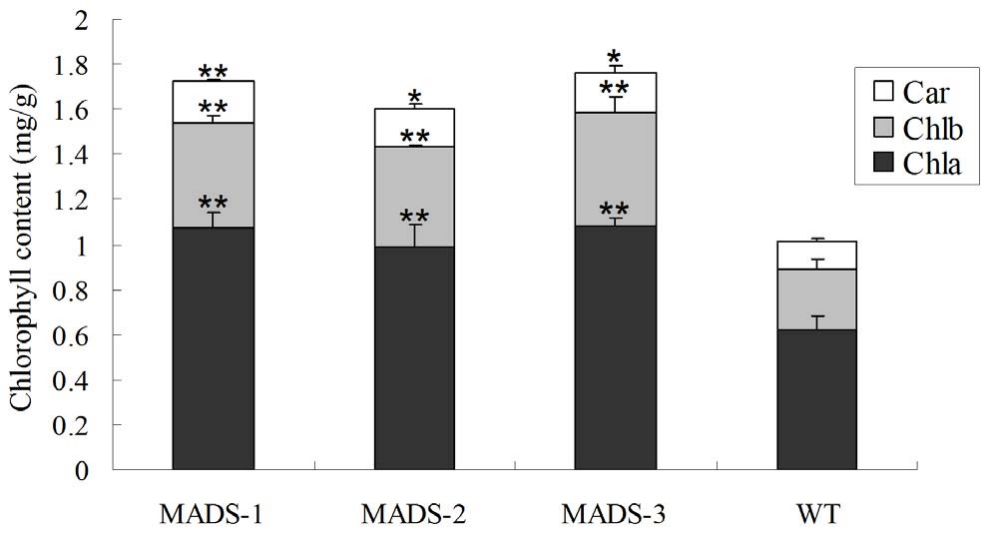
Chlorophyll content analysis. Leaves were collect from 25-day-old tobacco plants. Each transgenic line showed significant differences compared with wild-type, as determined by SPSS 11.5 analysis (Student's t-test, p  = 0.05); values are expressed as means (n  = 3 leaves for each test); error bars indicate S.D. ** p<0.01 for t test; * p<0.05 for t test. WT, wild-type; MADS1-3, 35S::BpMADS lines MADS1-3; Car, carotenoid; Chla, chlorophyll a; Chlb, chlorophyll b.

**Figure 8:**
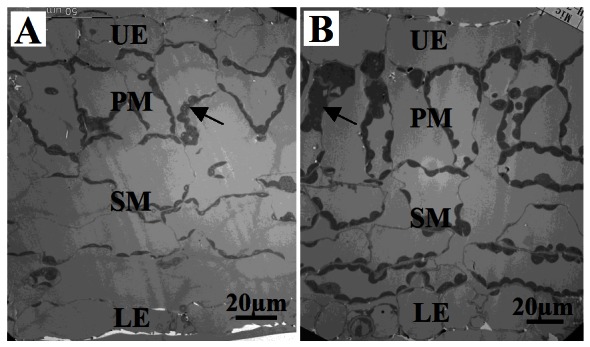
TEM observation of chloroplasts. For TEM observation of chloroplasts, second true leaves were collect from 14-day-old tobacco plants. (A) wild-type; (B) 35S::BpMADS line MADS-1. UE, upper-epidermis; PM, palisade mesophyll; SM, spongy mesophyll; LE, lower-epidermis. Arrows indicate chloroplasts.

**Figure 9:**
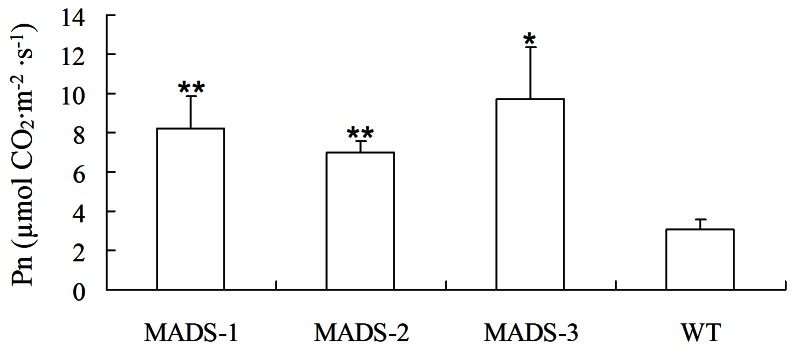
Photosynthetic rate analysis. Leaf photosynthesis was measured with a LI-6400 portable photosynthesis system. Data comparison between transgenic and wild-type was performed using SPSS 11.5 (Student's t-test, p  = 0.05); values are expressed as means (n  = 3 plant for each test); error bars indicate S.D. ** p<0.01 for t-test; * p<0.05 for t-test. WT, wild-type; MADS-1–3, 35S::BpMADS lines MADS-1–3.

**Figure 10:**
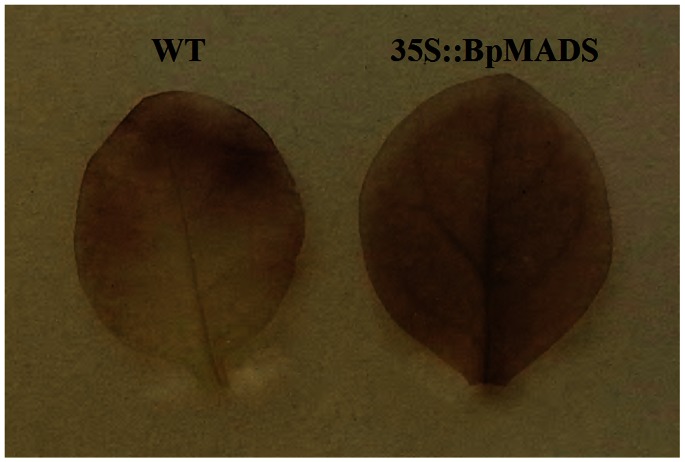
Starch-iodine staining of leaves. After 3 h of culture under normal light conditions, the leaves were removed from the plants and subjected to starch-iodine staining analysis.

The leaves of 35S::BpMADS plants were darker green than those of wild-type. Because chlorophyll gives leaves their green color, we examined the difference in chlorophyll content in wild-type vs. transgenic plants. The results show that there was a significant difference in both total chlorophyll content and the contents of pigments, including chlorophyll a, chlorophyll b, and carotenoid, between young 35S::BpMADS and wild-type plants (Figure 7). In addition, TEM also showed that the chloroplasts in both the palisade and spongy mesophyll cells of transgenic leaves were bigger and more abundant than those in wild-type leaves (Figure 8). Together, these results suggest that the overexpression of *BpMADS* enhances chlorophyll synthesis as well as chloroplast division and growth. Chlorophyll is extremely important for photosynthesis in plants. We therefore examined the photosynthetic rate of transformed and wild-type plants using a portable photosynthesis system. A high photosynthetic rate was detected in 35S::BpMADS plants, with a level more than 2-fold higher than that of wild-type plants (Figure 9). In addition, transgenic leaves exhibited stronger starch-iodine staining than wild-type (Figure 10).

### Grafting Experiment

**Figure 11:**
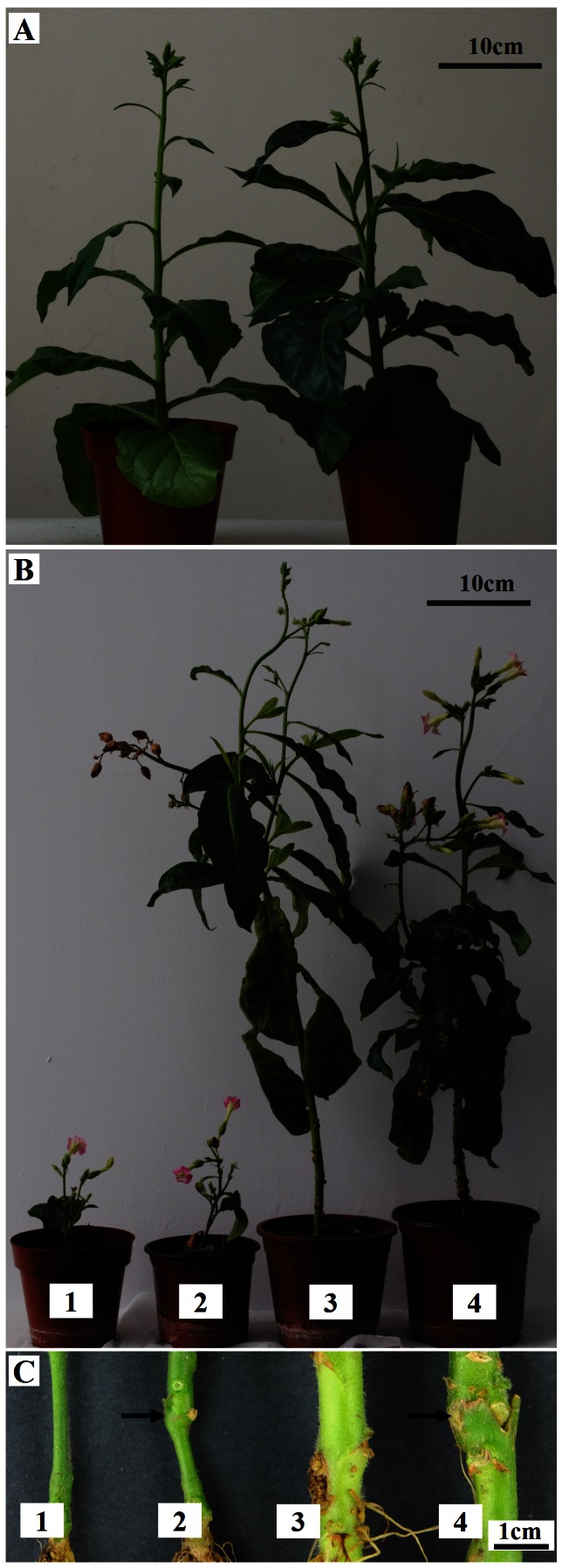
Grafting experiment. (A) The wild-type (left) and 35S::BpMADS stock+wild-type scion (right) plants were in the flowering stage. (B, C) 90-day-old plants (B) and a close-up view near the base of each stem (C). 1, 35S::BpMADS; 2, wild-type stock +35S::BpMADS scion; 3, wild-type; 4, 35S::BpMADS stock+wild-type scion.

Some floral stimuli, such as FT protein, act as long-distance signals that induce plant flowering. To examine whether BpMADS or a downstream factor can stimulate flowering, a grafting experiment was performed using 35S::BpMADS transgenic and wild-type tobacco plants. In this experiment, the genotype of the stock did not significantly affect flowering in the scion shoot (Figure 11). When transgenic stock was grafted to a wild-type scion shoot, the wild-type shoot flowered only when it reached the height at which conventionally grown wild-type shoots flower. However, the growth of the stock was synchronized to that of the scion shoot. Normally, the stem diameter of flowering plants is greater than 8 mm in the wild-type and less than 5 mm in the 35S::BpMADS plant. However, the 35S::BpMADS stock stem was greater than 7 mm in diameter due to the effects of the wild-type scion. On the contrary, the 35S::BpMADS scion inhibited stem growth in the wild-type stock; the stem of the wild-type stock was less than 4 mm in diameter. An examination of transverse sections of tobacco revealed that 35S::BpMADS tobacco stems had fewer secondary xylem cells than control stems (data not shown). Because the proliferation of secondary xylem cells results from long-range cell division in the vascular cambium, the enhanced flowering induced by the overexpression of *BpMADS* may have inhibited cell division in the vascular cambium, thereby retarding secondary xylem differentiation in tobacco. No *BpMADS* mRNA was detected in wild-type stock or wild-type scion by RT-PCR after 40 cycles of amplification (data not shown), suggesting that *BpMADS* mRNA could not move through the graft joint to the wild-type region of the grafted plant.

### Expression Levels of Flowering-related Genes

**Figure 12:**
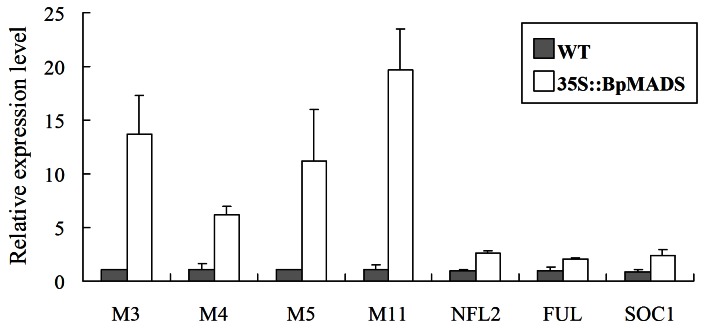
Relative expression levels of flowering-related genes. 35S::BpMADS plants showed significant differences in the relative expression levels of flowering-related genes compared with the wild-type, as determined by SPSS 11.5 analysis using Student's t-test (p<0.01). Values are expressed as means (n  = 3); error bars denote S.D. M3, *NsMADS3*; M4, *NtMADS4*; M5, *NtMADS5*; M11, *NtMADS11*; NFL2, *NFL2*; FUL, *NtFUL*; SOC1, *NtSOC1* were induced in transgenic plants.

Q-PCR was performed to estimate the transcript levels of flowering-related genes using the total RNA from aerial parts of 20-day-old transgenic and wild-type tobacco plants. Several reported flowering-related genes, such as *NsMADS3* (GenBank Accession No. AF068722), *NtMADS4* (No. AF068723), *NtMADS5* (No.AF068724), *NtMADS11* (No.AF385746), *NFL2* (No. U16174), *NtSOC1* (No. X76188), and *NtFUL* (No. DQ534202), were selected for analysis in transgenic 35S::BpMADS tobacco. As shown in Figure 12, the expression of all these genes was significantly upregulated in transgenic tobacco compared with the wild-type. Specially, the expression of *NsMADS3*, *NtMADS4*, *NtMADS5*, and *NtMADS11* increased more than 5-fold in transgenic tobacco compared with the wild-type. Because all of these flowering-related genes were significantly up-regulated in the transgenic lines, and transgenic stock overexpressing *BpMADS* did not improve the floral transition in the wild-type scion, it appears that the above flowering-related genes cannot activate transportable floral stimuli to affect the wild-type scion.

## Discussion

In plants, various signaling pathways regulate the expression of a relatively small number of common targets, which have been referred to as central floral pathway integrators or ‘‘integrator genes’ [1]. Different integrators directly or indirectly activate *AP1*, which marks the beginning of floral organ formation. In this study, we identified a MADS-box gene from birch, *BpMADS*, which is an ortholog of *AP1* in *Arabidopsis*. *BpMADS* exhibited some differences in expression compared with *AP1* of *Arabidopsis*. For example, C-terminal AP1-GFP appeared to be exclusively nuclear, while C-terminal BpMADS-GFP produced both cytoplasmic and nuclear signals. The results also indicate that the overexpression of *BpMADS* significantly promotes the flowering of transgenic tobacco seedlings. These results are consistent with those of Elo et al. [17].

When transgenic leaf disks were cultured on differentiation medium under normal light conditions, normally indeterminate shoot apices developed into terminal rudimentary flowers (Figure 5). Also, some flower buds formed directly from transgenic leaf disk on differentiation medium under normal tissue culture conditions, with an absence of the vegetative phase. Because the leaf is a typical plant organ consisting of differentiated cells, our results suggest that some well-differentiated cells of plant organs can directly redifferentiate into floral meristems. A similar phenotype is also found in embryonic flower (emf) mutants, which are characterized by the absence of rosette growth and floral structures that develop directly from embryos or calli [21], [22]. Our results also suggest that the acceleration of the floral transition caused by the overexpression of *BpMADS* in tobacco absolutely requires the presence of light. Besides the overexpression of *BpMADS* caused earlier flowering in tobacco, other pleiotropic effects of the overexpression of *BpMADS* were also examined. The transgenic tobacco exhibited accelerated development of lignin, and a higher ratio of leaf length/width. These characteristics are indicators of the transition from the vegetative to the reproductive phase that are commonly seen in the wild-type (*Nicotiana tabacum* cv. ‘Havana SRI’). Perhaps the transgenic tobacco exhibited early signs of flowering.

One of the most striking features of 35S::BpMADS tobacco plants is the enhanced chloroplast development in the leaves. We also found that 35S::BpMADS birch contains more chlorophyll than wild-type birch (data unpublished). The chloroplast is crucial for the process of photosynthesis, which captures light energy and stores it as chemical energy in the form of carbohydrate molecules. The development of normal chloroplasts is a crucial step in plant growth and development. For example, mutations in the chloroplast-localized *Thf1* and *AtTerC* genes, both of which are required for the structure of the thylakoid, can inhibit the development of *Arabidopsis* plants [23], [24]. Because higher chlorophyll levels were detected in 35S::BpMADS tobacco than in the wild-type, it is not surprising that 35S::BpMADS tobacco plants exhibited much higher photosynthetic rates than wild-type, which was also confirmed by histochemical staining for starch.

The 35S::BpMADS tobacco plants have developed several strategies to increase their photosynthetic rates compared with wild-type plants, including the following: (i) higher chlorophyll content; (ii) larger palisade cells; and (iii) enhanced guard cell development. These strategies help the plant capture light energy and improve gas exchange to make photosynthesis easier, thus producing more chemical energy for the plant. In flowering 35S::BpMADS plants, the flower and fruits comprised more than one fifth of the total fresh weight of the plant. However, the transgenic fruit matured normally, probably due to the high efficiency of photosynthesis, which provided adequate levels of carbohydrate for fruit development. Because *BpMADS* is expressed at extremely low levels in the young leaves of birch, which contain chloroplasts that undergo division, we propose that *BpMADS* is not required for chloroplast division in birch. This notion is also supported by the fact that no significant difference in chloroplast content was found between the *ap1-10* mutant (seed stock No. CS6230 in TAIR) and wild-type *Arabidopsis*. However, perhaps the high chloroplast content in 35S::BpMADS tobacco is a byproduct of the overexpression of *BpMADS*. To date, no other report has addressed the relationship between chloroplasts and AP1.

The FT protein is transported to the apical meristem [25]–[27], where it interacts with other factors, finally leading to the activation of floral meristem identity genes and the formation of flowers. To address whether BpMADS or other flowering-related factors that interact with BpMADS can move through the plant or activate transportable floral stimuli, a grafting experiment was performed. In the present study, no transportable *BpMADS* mRNA was detected in wild-type scion or stock, and the transgenic stock did not significantly enhance the flower transition of wild-type scion. In addition, wild-type stock did not attenuate the accelerated flowering induced by the overexpression of *BpMADS* in transgenic scion. These results suggest that flowering time in the scion is not affected by the presence or absence of 35S::BpMADS in the stock. AP1 in *Arabidopsis*, and its orthologs in other plant species, are meristem-identity genes, which act to control the initiation of flowering. The present results also confirm that the overexpression of *BpMADS* in leaves cannot accelerate the floral transition of the meristem. Perhaps the overexpression of *BpMADS* in the SAM promotes the floral meristem conversion in 35S::BpMADS tobacco seedlings. Moreover, *BpMADS* overexpression induced direct flower formation from transgenic tobacco leaves *in vitro*.

Several studies have shown that the ectopic expression of some MADS-box genes induces early flowering and enhances the expression of endogenous flowering-related genes in tobacco [28], [29]. In the present study, the expression of endogenous *NsMADS3*, *NtMADS4*, *NtMADS5*, *NtMADS11*, *NFL2*, *NtSOC1*, and *NtFUL* were induced in transgenic plants. Previous reports have also shown that ectopic expression of *NtSOC1*, *NtFUL*, *NsMADS3*, *NtMADS4*, or *NsMADS2* (ortholog of *NtMADS5*) accelerates the transition to flowering in tobacco, and ectopic expression of *NtMADS11* rescues the floral organ defect in the *ap1-1* mutant of *Arabidopsis* [28], [30], [31]. All of the flowering-related genes examined in the current study exhibit increased expression in floral organs and play important roles in the floral transition in tobacco. Perhaps the increased expression of these genes in transgenic tobacco resulted from the earlier floral transition that resulted from *BpMADS* overexpression. However, the floral transition occurs via strict cooperation between many floral factors. Each flowering-related gene exhibits a different expression profile. For example, *AP1* is only localized to sepals and petals in *Arabidopsis*, whereas ectopic expression of *AP1* in *Arabidopsis* induces early flowering with normal flowers, suggesting that some flowering-related genes function together to convert shoots into flowers.

## References

[1] Srikanth A, Schmid M (2011) Regulation of flowering time: all roads lead to Rome. Cell Mol Life Sci 68: 2013–2037. 21611891 10.1007/s00018-011-0673-y PMC11115107

[2] Reeves PH, Coupland G (2001) Analysis of flowering time control in *Arabidopsis* by comparison of double and triple mutants. Plant Physiol 126: 1085–1091. 11457959 10.1104/pp.126.3.1085 PMC116465

[3] Bernier G, Périlleux C (2005) A physiological overview of the genetics of flowering time control. Plant Biotechnol J 3: 3–16. 17168895 10.1111/j.1467-7652.2004.00114.x

[4] Mouradov A, Cremer F, Coupland G (2002) Control of flowering time: interacting pathways as a basis for diversity. Plant Cell 14: S111–S130. 12045273 10.1105/tpc.001362 PMC151251

[5] Mandel MA, Yanofsky MF (1995) A gene triggering flower formation in *Arabidopsis* . Nature 377: 522–524. 7566148 10.1038/377522a0

[6] Weigel D, Nilsson O (1995) A developmental switch sufficient for flower initiation in diverse plants. Nature 377: 495–500. 7566146 10.1038/377495a0

[7] Bowman JL, Alvarez J, Weigel D, Meyerowitz EM, Smyth DR (1993) Control of flower development in *Arabidopsis thaliana* by *APETALA1* and interacting genes. Development 119: 721–743.

[8] Ng M, Yanofsky MF (2001) Activation of the *Arabidopsis* B class homeotic genes by *APETALA1* . Plant Cell 13: 739–753. 11283333 10.1105/tpc.13.4.739 PMC135542

[9] Livak K J, Schmittgen TD (2001) Analysis of relative gene expression data using real-time quantitative PCR and the 2^−ΔΔ*C*^ _T_ Method. Methods 25: 402–408. 11846609 10.1006/meth.2001.1262

[10] Clough SJ, Bent AF (1998) Floral dip: a simplified method for Agrobacterium-mediated transformation of *Arabidopsis thaliana* . Plant J 16: 735–743. 10069079 10.1046/j.1365-313x.1998.00343.x

[11] Niwa Y (2003) A synthetic green fluorescent protein gene for plant biotechnology. Plant Biotechnol (Japanese journal) 20: 1–11.

[12] Chen H, Nelson RS, Sherwood JL (1994) Enhanced recovery of transformants of *Agrobacterium tumifaciens* after freeze-thaw transformation and drug selection. Biotechniques 16: 664–668. 8024787

[13] Horsch RB, Fry JE, Hoffmann NL, Eichholtz D, Rogers SG, et al (1985) A simple and general method for transferring genes into plants. Science 227: 1229–1231. 17757866 10.1126/science.227.4691.1229

[14] Jefferson RA, Kavanagh TA, Bevan MW (1987) GUS fusions: β-glucuronidase as a sensitive and versatile gene fusion marker in higher plants. EMBO J 6: 3909–3907. 3327686 10.1002/j.1460-2075.1987.tb02730.x PMC553867

[15] Barnes JD, Balaguer L, Manrique E, Elvira S, Davison AW (1992) A reappraisal of the use of DMSO for chlorophylls a and b in lichens and higher plants. Environ Exp Bot 32: 85–100.

[16] Shinano T, Lei TT, Kawamukai T, Inoue MT, Koike T, et al (1996) Dimethylsulfoxide method for the extraction of chlorophylls a and b from the leaves of wheat, field bean, dwarf bamboo, and oak. Photosynthetica 32: 409–415.

[17] Elo A, Lemmetyinen J, Turunen ML, Tikka L, Sopanen T (2001) Three MADS-box genes similar to APETALA1 and FRUITFULL from silver birch (*Betula pendula*). Physiol Plant 112: 95–103. 11319020 10.1034/j.1399-3054.2001.1120113.x

[18] Wu X, Dinneny JR, Crawford KM, Rhee Y, Citovsky V, et al (2003) Modes of intercellular transcription factor movement in the *Arabidopsis* apex. Development 130: 3735–3745. 12835390 10.1242/dev.00577

[19] Mandel MA, Gustafson-Brown C, Savidge B, Yanofsky MF (1992) Molecular characterization of the *Arabidopsis* floral homeotic gene *APETALA1* . Nature 360: 273–277. 1359429 10.1038/360273a0

[20] Pomar F, Merino F, Ros Barceló A (2002) O-4-Linked coniferyl and sinapyl aldehydes in lignifying cell walls are the main targets of the Wiesner (phloroglucinol-HCl) reaction. Protoplasma 220: 17–28. 12417933 10.1007/s00709-002-0030-y

[21] Sung ZR, Belachew A, Shunong B, Bertrand-Garcia R (1992) *EMF*, an *Arabidopsis* Gene Required for Vegetative Shoot Development. Science 258: 1645–1647. 17742537 10.1126/science.258.5088.1645

[22] Yang CH, Chen LJ, Sung ZR (1995) Genetic regulation of shoot development in *Arabidopsis*: role of the *EMF* genes. Dev Biol 169: 421–435. 7781888 10.1006/dbio.1995.1158

[23] Wang Q, Sullivan RW, Kight A, Henry RL, Huang J, et al (2004) Deletion of the chloroplast-localized Thylakoid formation1 gene product in *Arabidopsis* leads to deficient thylakoid formation and variegated leaves. Plant Physiol 136: 3594–3604. 15516501 10.1104/pp.104.049841 PMC527158

[24] Kwon KC, Cho MH (2008) Deletion of the chloroplast-localized *AtTerC* gene product in *Arabidopsis thaliana* leads to loss of the thylakoid membrane and to seedling lethality. Plant J 55: 428–442. 18429937 10.1111/j.1365-313X.2008.03523.x

[25] Lifschitz E, Eviatar T, Rozman A, Shalit A, Goldshmidt A, et al (2006) The tomato FT ortholog triggers systemic signals that regulate growth and flowering and substitute for diverse environmental stimuli. Proc Natl Acad Sci USA 103: 6398–403. 16606827 10.1073/pnas.0601620103 PMC1458889

[26] Corbesier L, Vincent C, Jang S, Fornara F, Fan Q, et al (2007) FT protein movement contributes to long-distance signalling in floral induction of *Arabidopsis* . Science 316: 1030–1033. 17446353 10.1126/science.1141752

[27] Tamaki S, Matsuo S, Wong HL, Yokoi S, Shimamoto K (2007) Hd3a protein is a mobile flowering signal in rice. Science 316: 1033–1036. 17446351 10.1126/science.1141753

[28] Smykal P, Gennen J, De Bodt S, Ranganath V, Melzer S (2007) Flowering of strict photoperiodic Nicotiana varieties in non-inductive conditions by transgenic approaches. Plant Mol Biol 65: 233–242. 17660946 10.1007/s11103-007-9211-6

[29] Ma G, Ning G, Zhang W, Zhan J, Lv H, et al (2011) Overexpression of Petunia SOC1-like gene *FBP21* in tobacco promotes flowering without decreasing flower or fruit quantity. Plant Mol Biol Rep 29: 573–581.

[30] Jang S, Hong MY, Chung YY, An G (1999) Ectopic expression of tobacco MADS genes modulates flowering time and plant architecture. Mol Cells 9: 576–986. 10672923

[31] Jang S, An K, Lee S, An G (2002) Characterization of tobacco MADS-box genes involved in floral initiation. Plant Cell Physiol 43: 230–238. 11867703 10.1093/pcp/pcf015

